# Immune Enteral Nutrition Can Improve Outcomes in Medical-Surgical Patients with ARDS: A Prospective Randomized Controlled Trial

**DOI:** 10.4172/2161-0509.1000109

**Published:** 2012-03-26

**Authors:** Elamin M Elamin, Andrew C Miller, Sophia Ziad

**Affiliations:** 1 James A. Haley Veterans Hospital, Divisions of Pulmonary & Critical Care Medicine, 13000 Bruce B. Downs Blvd. (111C), Tampa, FL 33612, USA; 2 Department of Internal Medicine, and Divisions of Pulmonary & Critical Care Medicine, University of South Florida, Tampa, FL, USA; 3 Department of Critical Care Medicine, National Institutes of Health, Bethesda, MD, USA; 4 Division of Pulmonary, Allergy and Critical Care Medicine, University of Pittsburgh Medical Center, Pittsburgh, PA, USA; 5 Department of Mathematics and Statistics, University of Maryland, Baltimore, Maryland, USA

**Keywords:** Acute respiratory distress syndrome (ARDS), Eicosapentaenoic acid, Gamma-linolenic acid, Enteral nutrition, Antioxidant, Multiple organ dysfunction

## Abstract

**Objective:**

To determine if early continuous enteral feeding of a diet containing eicosapentaenoic acid (EPA), gamma-linolenic acid (GLA), docosahexaenoic acid, and antioxidants in surgical-medical patients with ARDS improves Lung Injury Score (LIS), gas exchange, Multiple Organ Dysfunction (MOD) Score, ICU length of stay, and days on mechanical ventilation.

**Methods:**

Prospective randomized 2-center double-blind controlled trial of 17 ARDS patients whom continuously tube-fed the experimental diet (n=9) or an isonitrogenous, isocaloric standard diet (n=8) at a minimum caloric delivery of 90% of basal energy expenditure.

**Results:**

In the experimental group, there was a decrease in lung injury score (*p* < 0.003) and lower ventilation variables (*p* < 0.001). Patients in the experimental group had a statistically significant decrease in 28-day MOD score (*p* < 0.05). The length of ICU stay was significantly decreased in the experimental group (12.8 vs. 17.5 days; *p* = 0.01). The study was underpowered to detect any survival benefits between the two groups.

**Conclusion:**

An EPA and GLA supplemented diet contributes to improved gas exchange in addition to decrease LIS, MOD scores and length of ICU stay in patients with ARDS. An EPA+GLA-enriched enteral diet may be an effective tool in the medical management of ARDS.

## Background

Acute respiratory distress syndrome (ARDS) is a complex multifactorial illness manifest clinically as refractory hypoxemia and pulmonary edema. Nearly 200,000 new cases of ARDS occur annually in US with a mortality rate of 32–45% [1]. It may be precipitated by acute inflammatory disorders secondary to trauma, burns, sepsis, pneumonia, pneumonitis or inhalation lung injury. Alterations in lung function are evident during early systemic responses to inflammatory effectors including complement activation and release and modulation of eicosanoids [2]. These events promote macrophage and neutrophil (PMN) accumulation and migration into the alveolar space, resulting in endothelial and alveolar damage by lysosomal contents and toxic oxygen species [3–8].

**Figure 1:**
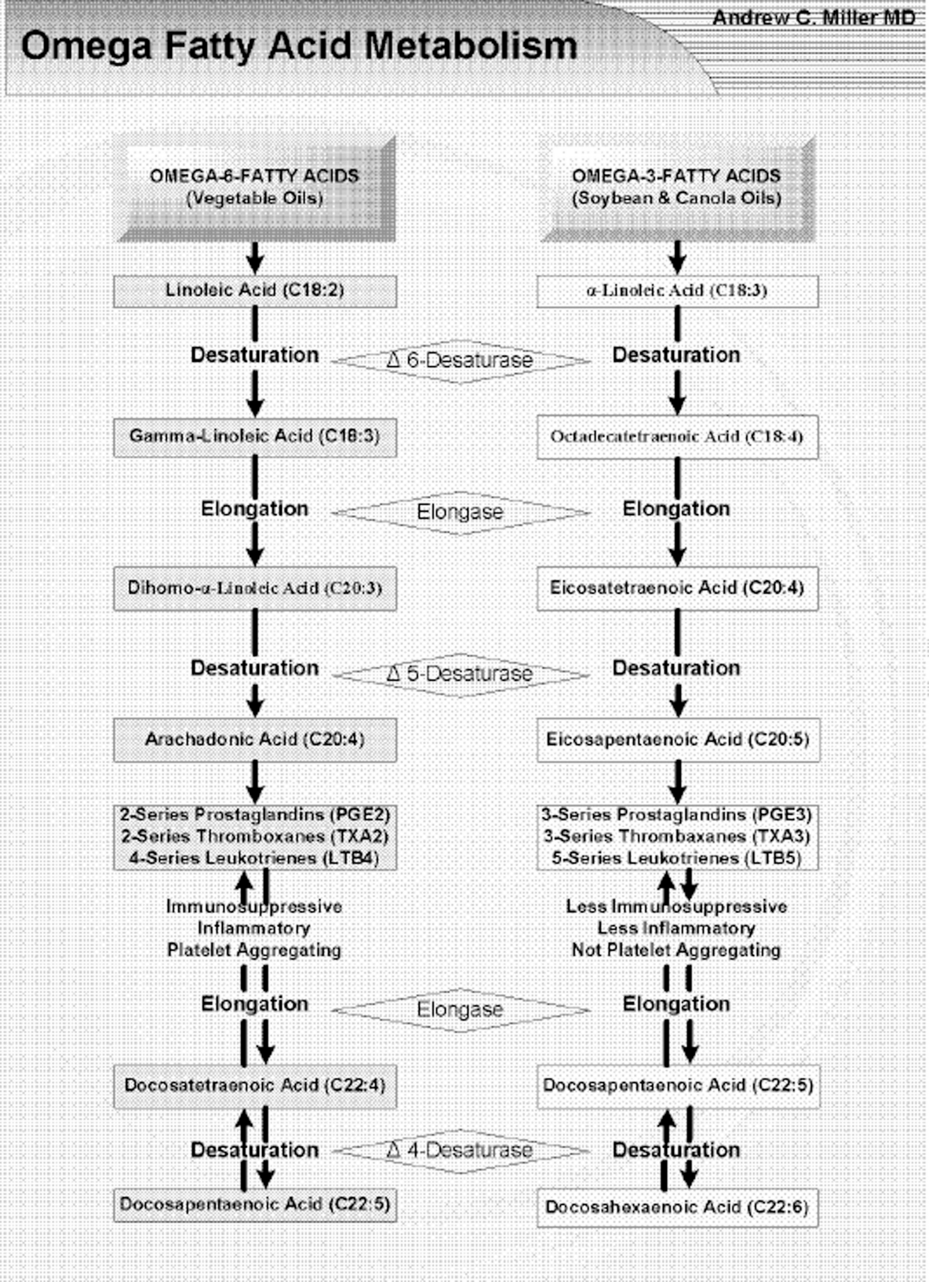
The metabolism of Omega-3- and Omega-6-Fatty Acids.

Dietary fatty acids may be oxidized for energy, stored in adipose tissue, or further metabolized to various long-chain polyunsaturated fatty acids (PUFA). Membrane derived PUFA’s serve as substrates for formation of eicosanoid effectors (omega-3 and omega-6) of the inflammatory response to infection or injury, Figure 1 [9]. Arachadonic acid (AA) derived Thromboxane A_2_ promotes vasoconstriction and platelet aggregation [10]. While, Leukotrienes C_4_, D_4_ and E_4_ generated by 5-lipoxygenase induce arteriolar constriction and increase post-capillary permeability, leading to edema [10–13]. In the clinical setting, the over production and release of these cell effectors appear to be involved in the etiology of ARDS [14].

Experimental and clinical studies have shown that the host’s immune response to a given stimulus can be down regulated by nutritional pretreatment with diets enriched with fish oil fatty acids including eicosapentaenoic (EPA) and docosahexaenoic (DHA) acids [15]. Similarly, attention has focused on borage seed oils containing relatively large amounts of gamma-linoleic acid (GLA) (18:3n6) which may be converted to dihomo-gammalinoleic acid (DGLA) (20:3n6) [16,17]. In a Brazilian study, amongst 165 patients with ARDS, those treated with a standard diet supplemented with EPA + GLA had significantly lower 28-day mortality, improved oxygenation status, more ventilator-free days, and lesser development of new organ dysfunction [18]. This was partially supported by an Israeli study which noted that amongst 100 ARDS patients, those treated with a standard diet supplemented with EPA + GLA showed significant improvement in oxygenation, lung compliance, and shorter ICU stays, however no statistical difference in survival was observed [19].

## Methods

The protocol was a prospective, randomized controlled double-blind study in mixed surgical-medical intensive care unit (ICU) at two tertiary-care academic teaching hospitals in the United States over 18-months period (Table 1). Critically ill patients with a diagnosis of a condition resulting in ARDS and meeting inclusion criteria were considered for enrollment in the study and randomized into one of two the treatment groups. Inclusion criteria were age 18–80 years old; condition resulting in ARDS as defined by the American-European Consensus Conference [20]; endotracheal and enteral feeding tubes in place; Modified Lung Injury Scores (LIS) > 2.5; and multiple organ dysfunctions (MOD) score < 9. The lung injury score (LIS) was calculated by averaging the chest roentgenogram score, PaO_2_/FIO_2_ score, PEEP score, and respiratory system compliance score as proposed by Murray et al. (Table 2) [21]. The multiple organ dysfunction score (MOD), constructed using simple physiologic measures of dysfunction in six organ systems (Table 4) [22], mirrors organ dysfunction as the intensivist sees it and correlates strongly with the ultimate risk of ICU mortality and hospital mortality, Table 3. Patients were excluded if any of the following were present: left ventricular heart failure defined as pulmonary capillary wedge pressure > 18 mmHg or left ventricular ejection fraction < 40%.; lung cancer (primary or metastatic); acute lymphoblastic leukemia; active gastrointestinal bleed: immune suppression such as recent chemotherapy, prednisone more than 0.25 mg.Kg^−1^.day^−1^ or HIV disease; Glasgow Coma Scale < 5 secondary to head trauma; pregnancy; and admission MOD score > 9.

Patients with a clinical diagnosis of ARDS were enrolled by the study nurses who determined their APACHE III [23] score and randomly assigned each patient a number dedicating them to either the experimental (Oxepa^®^ Ross Labs, Chicago, Illinois, USA) or control (Pulmocare^®^ Ross Labs, Chicago, Illinois, USA) enteranl formula on an alternating basis, Table 2. She retained the only copies of the full randomization schedule that were kept in a locked safe. A pharmacist blinded to the patient’s clinical condition prepared the diet according to the number assigned to the patient. The treating physicians, caregivers, patients and families remained blinded to the diet selection at all times. Resting Energy Expenditure (REE) was calculated by the Weir formula [24] on enrollment day after measuring oxygen consumption and carbon dioxide production by the Deltatrac II calorimeter (Datex Instrumentation, Helsinki, Finland; VCO_2_-Deltatrac)[25].

The enteral diet was then delivered to all enrolled patients via a nasogastric, nasoduodenal, nasojejunal, or jejunostomy tubes. The enteral nutrition was delivered at a constant rate to achieve at least 65% REE within the first 24 hours, and then advanced to at least 90% REE within 72 hours. The first day that the patient received enteral nutrition at 90% REE or more was considered as study Day 1.

Patients in both EPA+GLA and control groups were ventilated by Puritan-Bennett 7200 series ventilator (Puritan Bennett, Carlsbad, CA). All the study measurements were obtained every morning between 7:00 AM and 12:00 PM before any changes were made in the patient’s mechanical ventilation or oxygen delivery settings.

Enteral feeding with the EPA+GLA diet was continued for 7 days at that point enteral nutrition was converted to the standard feeding formula selected by the treating physician. Patients received enteral feeding until able to tolerate oral intake. However, daily evaluation of various clinical parameters continued until discharge from the ICU, then weekly until hospital discharge.

## Outcome Measures

Primary outcome measures included oxygenation and modified Lung Injury Scores (LIS) assessed at days 1, 4, and 7. While secondary outcome measures included, Multiple Organ Dysfunction (MOD) scores, days on mechanical ventilation and total length of ICU stay.

## Statistics

Patients were analyzed on an intention-to-treat basis. Statistical variables are expressed as mean values, standard deviations and medians as appropriate. Survival analysis was assessed using SAS Life Test (© SAS Institute Inc., Cary, North Carolina). When appropriate, mixed modeling was used to assess repeated measures.

Since repeated measures (respective variable) were found for each patient over a 7-day course, with respect to group, the multivariate mixed model analysis was applied to determine whether the measurements suggest significance within and between the experimental and control groups. In addition, Student’s T-test was utilized for comparing the baseline values between the two groups. All *p* values were two-sided and significance was assigned at a threshold *alpha* of 0.05.

## Ethics

The institutional review board at Southern Illinois University School of Medicine in addition to the review boards at Memorial Medical Center and Saint John’s Hospital in Springfield, Illinois approved the protocol. A written informed consent was obtained from each patient or health care proxy before the enrollment of every study subject. Both principal investigators and the clinical coordinator were available 24-hours-per-day throughout the study to answer questions regarding safety, patient eligibility, and for reporting of any adverse events.

## Results

**Figure 2:**
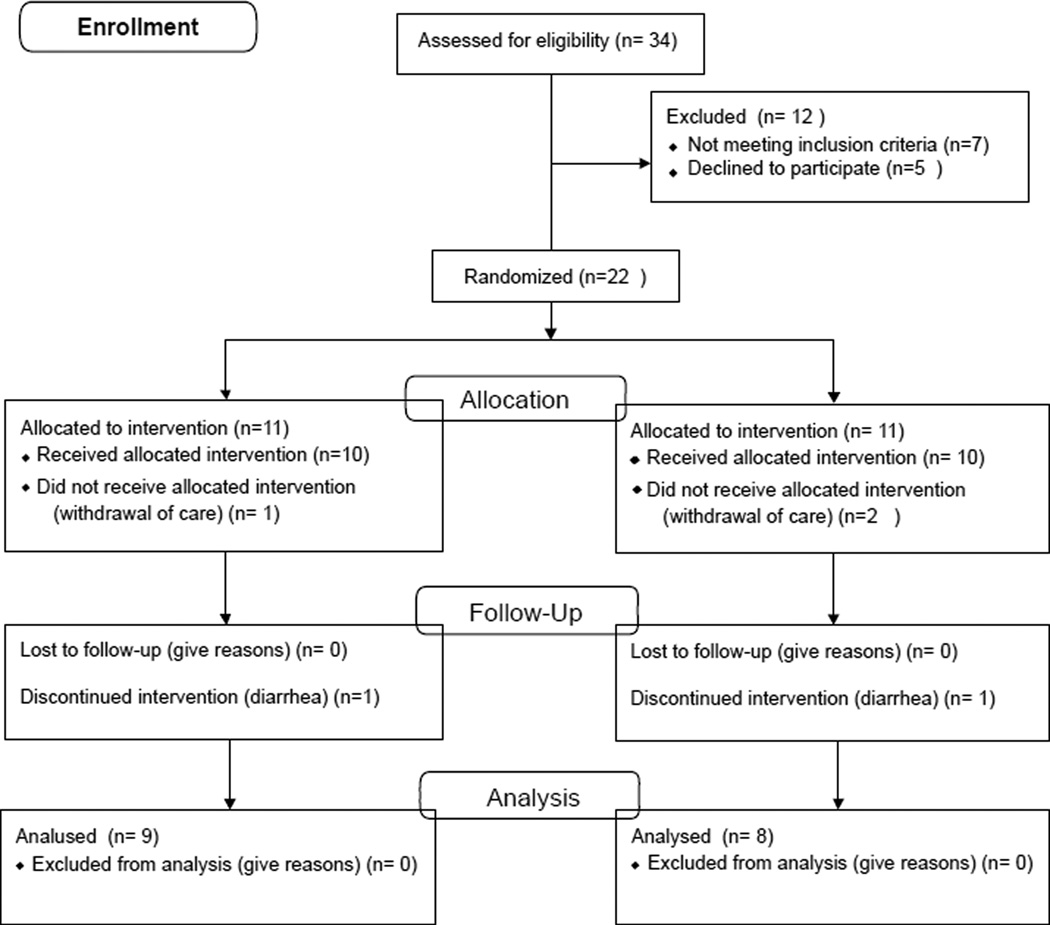
Study flow diagram.

The analysis was conducted in 17 out of 22 patients who were enrolled in the study. Reasons for exclusion from analysis was diarrhea (n=1) and withdrawal of care (n=3), see Figure 2. There were no significant differences between the two groups regarding age, gender; ICU admission diagnosis and APACHE III score at enrollment, Table 4. There was no significant difference in time from hospital admission to study entry between the study group (6.7 ± 2.3 days) and the control group (8.2 ± 3.5) (*P*=0.3). In addition, time to achievement of 90% of REE and duration of tube feeding was similar between the two groups. All enrolled patients were fed successfully for two weeks through naso-gastric, naso-duodenal or naso-jejunal route.

## Primary outcome measures

### Oxygenation and modified lung injury scores (LIS) oxygenation

There were no statistically significant baseline differences between the two groups regarding PaO_2_, FiO_2_, or PaO_2_/FiO_2_ ratio. Applying a multivariate mixed model analysis, results for PaO_2_/FiO_2_ ratio in the EPA+GLA group were statistically significant through Day 2 (p < 0.01), and then stabilized by day 7 (Table 5).

In addition, the LIS was calculated on entry to the study, and then on study days 1, 4 and 7. Applying a multivariate mixed model analysis; the results demonstrated a statistically significant difference in the EPA+GLA group from Day 1 through Day 4 with (*p* < 0.003). In addition, there was a statistically significant decline in LIS in the EPA+GLA group on Day 7 comparing to pretreatment value, Table 6.

Furthermore, a comparison of ventilation variables on days 1, 4, and 7 are listed in Table 7. Although ventilation variables (FIO_2_, positive end-expiratory pressure, and minute ventilation) were similar between the two groups by day 7, lower ventilation variables were recorded in the EPA+GLA group patients with APACHE III scores >25 on admission to the ICU compared to the controls (*p* < 0.01).

### Secondary outcome measures

#### Multiple organ dysfunctions

**Figure 3:**
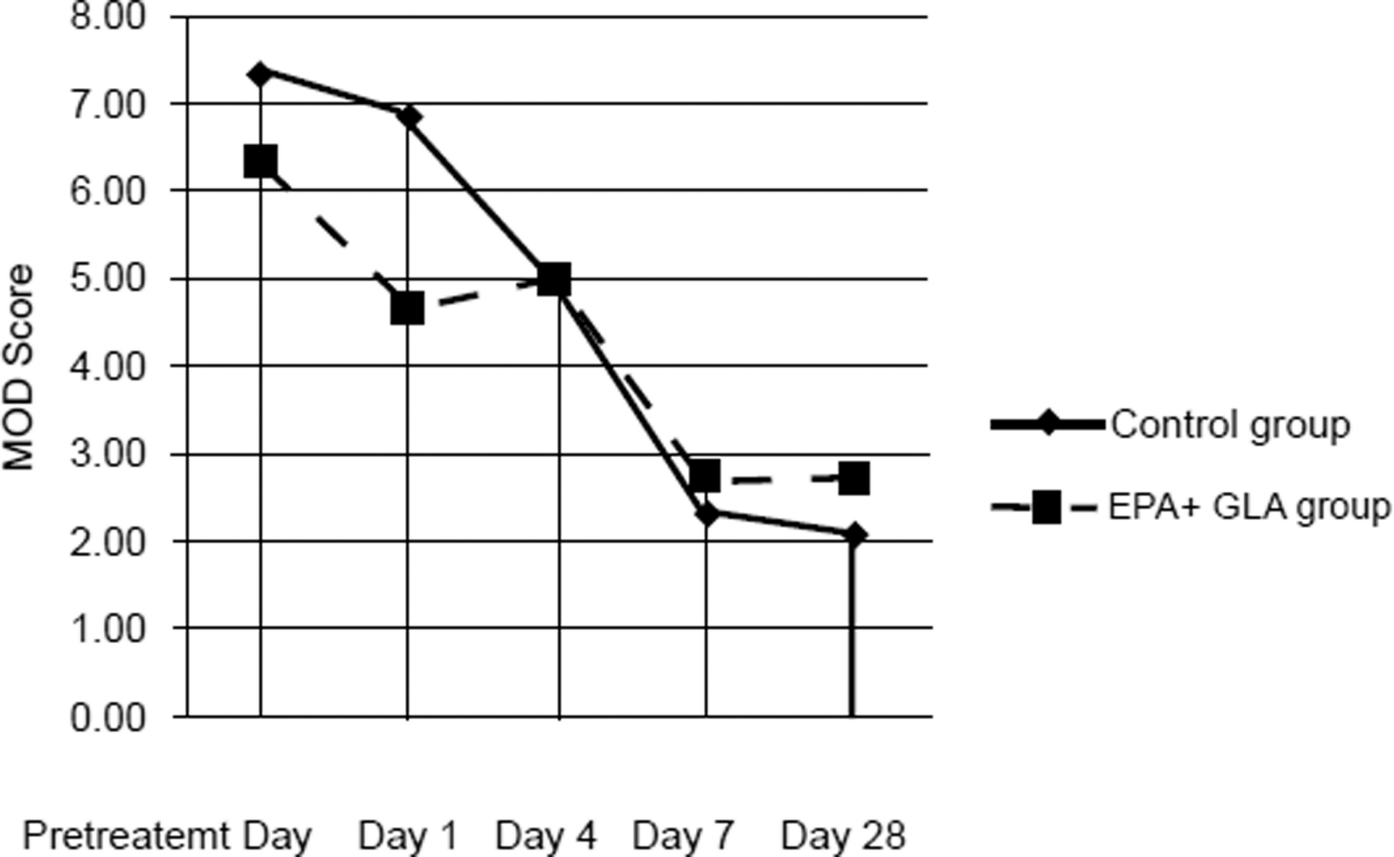
Effects of EPA and GLA enteral feedings on Multiple Organ Dysfunction (MOD) scores comparing to the control group.

We investigated the incidence of development of new organ dysfunction in both groups. By applying a multivariate mixed model analysis, the overall results demonstrated a trend toward lower MOD scores for the EPA+GLA group from Day 1 through 7 (p < 0.06) which was statistically remains significant by day 28 (p < 0.05), Figure 3.

#### Length of mechanical ventilation and ICU stay

**Figure 4:**
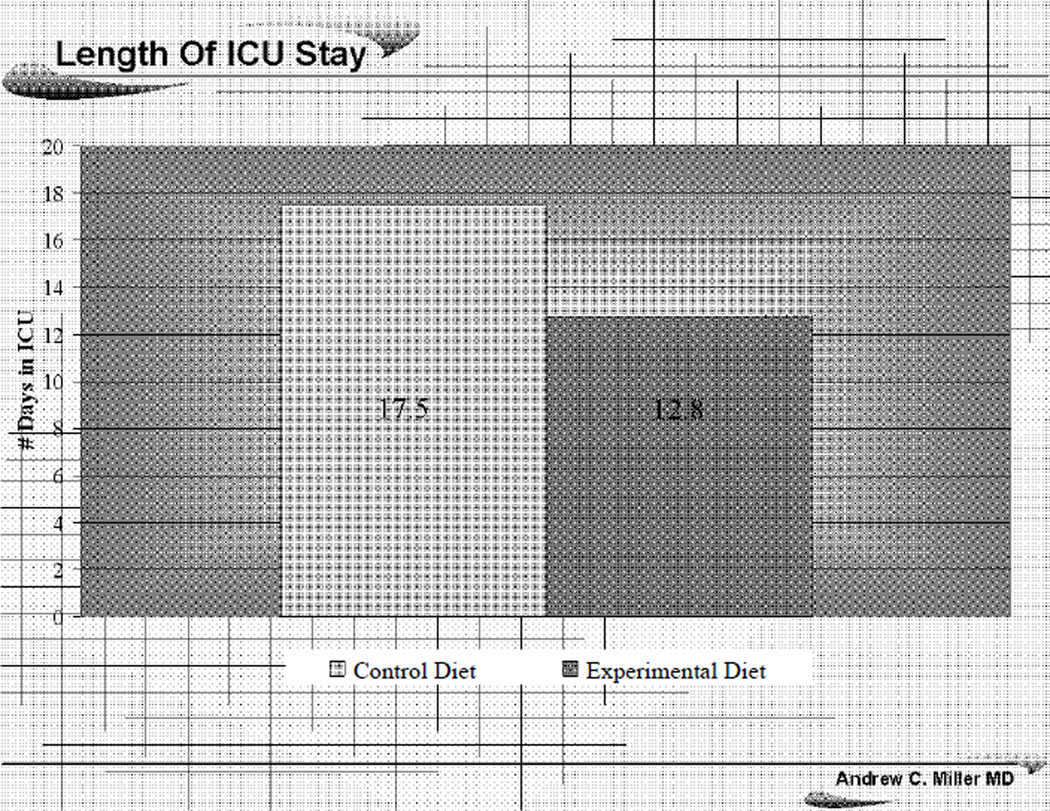
The effects of EPA+GPA diet on length of ICU stay comparing to the control group.

Patients fed the EPA+GLA diet supplemented with EPA, GLA, DHA, and antioxidants had a decreased length of stay in the ICU, 12.8 vs. 17.5 days for controls (*p* = .01), Figure 4. In addition, there was a trend towards a decreased number of ventilator dependant days in the EPA+GLA vs. control group (6.7 vs. 8.2), however these results failed to reach a statistical significance difference (*p* = 0.3).

Finally, although mortality was not an outcome measure, at the end of 28 days 0 of 9 patients died in the EPA+GLA group compared to 1 of 8 in the control group. However, such difference was not statistically significant, (*p*=0.3).

## Discussion

Early animal studies illustrated that diets supplemented with EPA and GLA may have beneficial effects in models of acute lung injury. A swine study suggested that EPA and GLA supplemented diets attenuated acute lung injury following endotoxin challenge [26,27].

Later human studies examined the immunologic effects of feeding ICU patients EPA, DHA, GLA and antioxidants supplemented diets [28,29]. The aim effects were changes on pulmonary inflammation, eicosanoid mediators, endogenous antioxidants, and pulmonary function in patients with acute pulmonary inflammation.

Other multi-center prospective randomized clinical trials examining the effects of early enteral administration of a formula supplemented with arginine, nucleotides, and fish oil in ICU patients found that such a strategy was safe, well tolerated and contributed to a significant reduction in hospital length of stay and the frequency of nosocomial infection [30].

Several studies investigated the role for EPA and GLA enhanced diets in a variety of patient populations [31–36]. In one such study, patients with ARDS or ALI fed an enteral diet supplemented with Oxepa enternal nutrition. The experimental group patients had reduced pulmonary neutrophil recruitment with improved oxygenation, significantly less number of ventilator-dependant days, shorter length of ICU stay, and decreased mortality compared to patients fed the control diet (Pulmocare) [31].

In our study, we expanded on the previously reported benefits of enteral diets enriched with Ω-3 and Ω-6 fatty acids and examined the effect of such a diet on the MOD score. Unlike number of the previous studies, we utilized the APACHE III score for objective comparison of the disease severity on entry to the study [18,19,31]. Furthermore, to maximize nutrient intake, we established enteral nutrition at 90% of REE within 72 hours of initiation of enteral feeding as compared to the 75% noted in another study [18].

In the present study, there was an early statistically significant difference in the PaO_2_/FiO_2_ ratio from Day 1 to Day 2 but then stabilized and became not significant for the remainder of the study. However, the LIS scores significantly improved in the EPA+GLA group from Day 1 to Day 4 with significant decline on Day 7 comparing to pretreatment value. Such finding again supports the better utility of the LIS comparing to the PaO_2_ or PaO_2_/FiO_2_ ratio in assessment of pulmonary response to different therapeutic interventions [37]. Another interesting findings in this study was that patients with higher enrollment APACHE III scores displayed the greatest benefits when fed EPA+GLA. The latter had lower FiO_2_ requirements, positive endexpiratory pressure, and minute ventilation requirements compared to controls (*p* < 0.01). This is somewhat similar to the findings from the PROWESS study, which showed that severe septic patients with the higher APACHE II scores, rather than APACHE III in our study had a lower mortality after treatment with recombinant activated protein C [38].

Another unique design feature of our study was the use of MOD for objective assessment of organs failure rather than relying on the percent of patients who developed a new organ failure in each group [31]. Overall, we found that patients who were fed EPA+GLA enriched diets had a moderately significant decrease in MOD score from days 1 through 7, reaching statistical significance by study day 28 (*p* < 0.05), Figure 3. These improvements in patient status translated into decreased length of ICU stay in the EPA+GLA fed group (12.8 vs. 17.5 days; *p* = *0* .01) compared to the control group, Figure 4. Considering the daily ICU expenditure in caring for ARDS patients, such decrease in length of ICU stay is expected to offset the cost of the EPA+ GLA enhanced enteral diet many fold. However, this study was not designed to assess that question and a larger pharmacoeconomics study with larger patient numbers is needed to investigate such issue

Finally, although mortality in both groups was recorded it was not one of the study outcome measures. In addition, the small number of patients enrolled in the current study precludes any solid conclusions to the survival benefits of the EPA+GLA enteral diet.

## Limitations

The small sample size is a limiting factor in this study. In the areas where we did not find statistical significance, it is possible that significance exists but the study was underpowered to detect it. However, the magnitude of the clinical advantage detected even with the small sample size may suggest efficacy of the intervention. Nevertheless, our data will still need further validation in a prospective independent sample of patients in a much larger multi-center study. The primary author of the present study is currently conducting a larger clinical trial to investigate the survival benefits of the EPA+GLA enteral diet in an independent sample of ICU patients.

## Conclusion

The findings of this non-pharmaceutically sponsored study support the previously reported benefits of an enteral diet supplemented with EPA, GLA, DHA and antioxidants on gas exchange and of length of ICU stay of ALI & ARDS patients. In addition, our study demonstrated that patients with higher APACHE III scores had additional benefit of decreasing 28-day MOD scores by receiving the EPA+GLA enteral diet. When one takes into account the low cost of this enteral therapy with its safety and minimal side effect profile, EPA+GLA-enriched enteral therapy may prove to be an integral component of our management strategy for ALI and ARDS. The latter is in agreement with the various scientific organizations clinical practice guidelines for nutrition support of mechanically, ventilated critically ill adult patients [39,40].

## Figures and Tables

**Table 1:** Comparison of study dietary regimens.

Parameter	Control Diet	Experimental Diet
**Caloric Distribution**		
% Protein	16.7	16.7
% Fat	55.2	55.2
% Carbohydrate	28.1	28.1
**Nutrient Sources**		
Protein	Sodium & calcium caseinates	Sodium & calcium caseinates
Fat	Corn oil	Canola oil, MCT, borage oil, fish oil
**Carbohydrate Sources**	Hydrolyzed cornstarch, Sucrose	Hydrolyzed cornstarch, Sucrose
**Other Specific Nutrients**	Ultra-trace minerals, High vitamin E, High vitamin C and β-carotene, Carnitine, Taurine	Ultra-trace minerals, High vitamin E, High vitamin C and β-carotene, Carnitine, Taurine
**Kilocalories/milliliter**	1.5	1.5
**Osmolality (mOSm/kg H_2_O)**	465	465
**Calorie-to-Nitrogen ratio**	150:1	150:1
mL/day to meet RDA*	947	947
**Form**	Ready to feed liquid	Ready to feed liquid

Both the control and experimental groups were matched with regard to percentage of protein, fat and carbohydrate in their respective diets as well as feed concentration (Kcal/ml), osmolality, and calore-to-nitrogen ratio.

* RDA: Recommended Daily Allowance.

**Table 2:** Components and individual values of the lung injury score.

Value			

1	Chest roentgenogram Score		
	No alveolar consolidation		0
	Alveolar consolidation confined to 1 quadrant		1
	Alveolar consolidation confined to 2 quadrants		2
	Alveolar consolidation confined to 3 quadrants		3
	Alveolar consolidation in all 4 quadrants		4

2	Hypoxemia score		
	PaO_2_/FIO_2_	≥300	0
	PaO_2_/FIO_2_	225–299	1
	PaO_2_/FIO_2_	175–224	2
	PaO_2_/FIO_2_	100–174	3
	PaO_2_/FIO_2_	<100	4

3	PEEP score (when ventilated)		
	PEEP	≥5 cm H_2_O	0
	PEEP	6–8 cm H_2_0	1
	PEEP	9–11 cm H_2_0	2
	PEEP	12–14 cm H_2_O	3
	PEEP	≥15 cm H_2_O	4

4	Respiratory system compliance score		
	Compliance	≥80 ml/cm H_2_0	0
	Compliance	60–79 ml/cm H_2_O	1
	Compliance	40–59 ml/cm H_2_0	2
	Compliance	20–39 ml/cm H_2_0	3
	Compliance	≤19 ml/cm H_2_0	4

The final value is obtained by dividing the aggregate sum by the number of components that were used.			
Score			
No lung injury- 0			
Mild-to-moderate lung injury −0.1–2.5			
Severe lung injury (ARDS) >2.5			

* Abbreviations: PaO_2_/FIO_2_ = Arterial oxygen tension to inspired oxygen concentration ratio; PEEP = Positive end-expiratory pressure.

**Table 3:** The Multiple Organ Dysfunction Score.

Organ System	Score				
	0	1	2	3	4

Respiratory a (PO_2_/FIO_2_ ratio)	>300	226–300	151–225	76–150	≤75

Renal b (serum creatinine)	≤100	101–200	201–350	351–500	>500

Hepatic c (serum bilirubin)	≤20	21–60	61–120	121–240	>240

Cardiovascular d (PAR)	≤ 10.0	10.1–15.0	15.1–20.0	20.1–30.0	>30.0

Hematologic e (Platelet count)	>120	81–120	51–80	21–50	≤20

Neurologic f (Glasgow Coma Score)	15	13–14	10–12	7–9	≤6

a The PO_2_/FIO_2_ ratio is calculated without reference to the use or mode of mechanical ventilation, and without reference to the use or level of positive end-expiratory pressure:

b The serum creatinine concentration is measured in µmol/L without reference to the use of dialysis;

c the serum bilirubin concentration is measured in µmol/L

d the pressure-adjusted heart rate (PAR) is calculated as the product of the heart rate (HR) multiplied by the ratio of the right atrial (central venous) pressure (RAP) to the mean arterial pressure (MAP): PAR= HR × RAP/ mean BP;

e the platelet count is measured in platelets/ml 10^−3^;

f the Glasgow coma Score is preferably calculated by the patient’s nurse, and is scored conservatively (for the patient receiving sedation or muscle relaxants, normal function is assumed, unless there is evidence of intrinsically altered mentation)

**Table 4:** Patient characteristics at time of enrollment.

Variables	Study (n=9)/ SD	Control (n=8) / SD	*p*

Age (years)	50.0 / 22.2	55.2 / 16.5	0.6

Gender			
Male	5	3	0.4
Female	4	5	0.6

APACHE III score	60.33 / 14.6	52.9 / 9.5	0.2

Diagnosis			
Medical	7	6	0.4
*Surgical (none trauma)	2	2	0.8

REE Kcal/day (Indirect Calorimetry)	1700 / 236	1681 / 294	0.3

LIS	2.83 / 0.28	2.84 / 0.51	0.9

MOD Score	7.33 / 1.4	6.12 / 2.0	0.3

Acute Physiology, Age, Chronic Health Evaluation (APACHE) III score, resting energy expenditure (REE), Lung Injury Score (LIS), and Multiple Organ Dysfunction (MOD) Score.

* Surgery prior to ICU admission

**Table 5:** Comparison of mean PaO2/FiO2 ratios on days 1, 2, 4 and 7.

Group	Day 1 PaO_2_/FIO_2_	Day 2 PaO_2_/FIO_2_	Day 4 PaO_2_/FIO_2_	Day 7 PaO_2_/FIO_2_
EPA+GLA	157	149	162	178
Control	138	142	145	201
P-value	0.01	0.12	0.38	0.11

**Table 6:** Comparison of mean Lung Injury Score from pretreatment day through study Day 7.

	Pretreatment Day	Study Day 1	Study Day 4	Study Day 7
Study group (n=9)	2.83±0.2	2.77±0.4	2.5±0.5	2.1±0.6*
Control group (n=8)	2.84±0.3	2.91±0.4	2.4±0.6	2.3±0.4

* Statistically significant decline comparing to pretreatment mean (P=0.04)

**Table 7:** Comparison of ventilation variables on days 1, 4, and 7.

Variable	Study Day	No.	Control (mean/ median)	No.	EPA+GLA (mean/ median)

FIO_2_	1	8	0.54 / 0.5	9	0.52 / 0.6
	4	8	0.53 / 0.48	7	0.57 / 0.45
	7	7	0.43 / 0.43	6	0.48 / 0.4

PEEP (cm H_2_O)	1	8	9.38 / 8	9	9.44 / 10
	4	8	8.63 / 8.5	7	8.29 / 8
	7	6	5.5 / 5	6	8.67 / 7.5

PIP (cm H_2_O)	1	8	31.38 / 30.5	9	28.11 / 29
	4	7	28.14 / 30	5	32.8 / 35
	7	5	28.2 / 28	5	35 / 31

PaO_2_ (mm Hg)	1	8	72.75 / 68.5	9	81.56 / 76
	4	7	87 / 78	7	92.29 / 89
	7	5	86.4 / 85	7	92.71 / 90

Minute Ventilation (L/min) *	1	8	9.76 / 8.82	9	11.94 / 10.5
	4	8	9.09 / 9.35	5**	16.03 / 17.6
	7	6	12.41 / 11.54	6	13.26 / 12.38

EPA: Eicosapentaenoic Acid; GLA: γ-linolenic acid; PEEP: Positive End-expiratory Pressure; PIP: Peak Inspiratory Pressure.

* Minute Ventilation (L/min) = (tidal volume in L) X (total ventilation rate in breaths/min)

** Data was missing for 1 patient.

## Abbreviations

ARDS: Acute Respiratory Distress Syndrome
ALI: Acute Lung Injury
AA: Arachadonic Acid
DHA: Docosahexaenoic Acid
DGLA: Dihomo-gammalinoleic Acid
EPA: Eicosapentaenoic Acid
GLA: Gamma-linolenic Acid
Ω-3: Omega-3
ICU: Intensive Care Unit
LT: Leukotriene
LA: Linoleic Acid
LOX: Lipoxygenase-derived hydroxyeicosatetraenoic acids (HETEs)
MOD: Multiple Organ Dysfunction
PMN: Neutrophil
PUFA: Polyunsaturated Fatty Acids
PG: Prostaglandin
TNF: Tumor Necrosis Factor
IL: Interleukin
REE: Resting Energy Expenditure
AM: Alveolar Macrophage
APACHE Score: Acute physiology, Age, Chronic Health Evaluation Score
RNA: Ribonucleic Acid
FO: Fish Oil
CO: Corn Oil
BO: Borage Oil
TX: Thromboxane
LIS: Lung Injury Scores (LIS)
